# Letter from J. H. Ridley, M. D., Huntsville, Ala.

**Published:** 1891-12

**Authors:** J. H. Ridley

**Affiliations:** Huntsville, Ala.


### Correspondence.

### A WOMAN'S LEG LITERALLY ROTTING OFF WITHOUT HEMORRHAGE; AND BONE SEPARATING WITHOUT ASSISTANCE OF PHYSICIAN.

HUNTSVILLE, ALA.

*Editors Atlanta Medical and Surgical Journal:*

Two years since an interesting case fell to me to treat, and I give you details. I was called in to see Mrs. James Bird, near Huntsville, Ala., and found her six months *enciente;* and two small spots on the inside of either limb. Patient complained of intense burning extending down from both spots and involving both feet and ankles. A peculiar diffusible red now set in from both spots, and patient had erysipelas in both limbs. I gave sulph. of quinia aud muriated tinct. of iron, and succeeded in curing the trouble in five days. The patient was so feeble and anemic I informed the husband of my apprehensions about the mother carrying the fetus to term. I placed her upon a tonic, and was call hastily in three weeks to the mother again. I found her in hard labor, and did not succeed in delivering in thirty-six hours. The two spots now returned in same place above the ankles on both limbs, and the erysipelas with redoubled vigor attacked both limbs, extending to within two inches of the knee. On the sixth day I discovered that gangrene had set in; the right side of the face, tongue and head turned black, and the whole system seemed permeated with the poison. I informed the husband of her great danger, and the patient said she preferred to die in peace rather than undergo operation. I did not think she could stand chloroform, and was confident the nervous shock would prove fatal. The line of demarkation between healthy flesh was well marked, and all the flesh dropped off from two inches below the knee to ankle, leaving the two bones bare and the ankles as black and dried up as an Egyptian mummy. She would not allow me to remove the limb and in attempting to turn herself in bed the bone separated two inches below the knee. There never was a particle of hemorrage, and the flesh gradually covered the bone, and the stump is a good one. There is no tenderness or feeling in the stump, and Mrs. Bird has grown fleshy and in better health than she ever enjoyed in her life.

Truly Nature is a wonderful restorer, and this case is so remarkable I concluded it ought to be reported.

The child lived three days, and the mother, always feeble before this trouble, now looks as if she would live to old age.

J. H. RIDLEY, M. D.

